# The novel multi-cytokine inhibitor TO-207 specifically inhibits pro-inflammatory cytokine secretion in monocytes without affecting the killing ability of CAR T cells

**DOI:** 10.1371/journal.pone.0231896

**Published:** 2020-04-22

**Authors:** Muneyoshi Futami, Keisuke Suzuki, Satomi Kato, Saori Ohmae, Yoshio Tahara, Masanori Nojima, Yoichi Imai, Takayuki Mimura, Yoshihiro Watanabe, Arinobu Tojo

**Affiliations:** 1 Division of Molecular Therapy, Advanced Clinical Research Center, The Institute of Medical Science, The University of Tokyo, Tokyo, Japan; 2 Research laboratories, Torii Pharmaceutical., Sakura-shi, Japan; 3 Center for Translational Research, The Institute of Medical Science Hospital, The University of Tokyo, Tokyo, Japan; 4 Department of Hematology/Oncology, The Institute of Medical Science, The University of Tokyo, Tokyo, Japan; 5 Innovative Clinical Research Center, Kanazawa University, Kanazawa, Japan; Texas Woman's University, UNITED STATES

## Abstract

Cancer immunotherapy using chimeric antigen receptor–armed T (CAR T) cells have been shown to improve outcomes significantly in patients with hematological malignancies. However, cytokine release syndrome (CRS) remains a risk. CRS is characterized by the excessive activation of CAR T cells and macrophages. Signs and symptoms of CRS are usually resolved after steroid administration, but steroids abrogate the expansion and persistence of CAR T cell populations. Tocilizumab is a humanized monoclonal antibody (mAb) that attenuates CRS without significant loss of CAR T cell activity. However, interleukin-6 (IL-6)/IL-6 receptor (IL-6R) blockade alone cannot relieve CRS symptoms fully, and novel treatments are needed to prevent or cure CRS. TO-207 is an N-benzoyl-L-phenylalanine derivative that significantly inhibits inflammatory cytokine production in human monocyte and macrophage-specific manner. We investigated whether TO-207 could inhibit cytokine production without impairing CAR T cell function in a CRS-simulating co-culture system.

## Introduction

Treatment with chimeric antigen receptor (CAR)-T cells has emerged as a promising therapeutic approach for cancer therapy. These engineered CAR T cells carry single-chain variable fragments (scFvs) that specifically bind to molecules expressed on the cell surfaces of cancer cells, as well as cytoplasmic T cell receptor (TCR) CD3ζ chain, and costimulatory receptors including CD28 and 4-1BB [1]. CAR T cells targeting CD19 are already used in clinical practice for the treatment of B-cell malignancies [2–6]. However, cytokine release syndrome (CRS), a life-threatening adverse event, is often observed in patients undergoing CAR T-cell therapy; CRS typically manifests as high fever, hypotension, hypoxia, and multiorgan failure [7]. Furthermore, CRS can progress into fulminant macrophage activation syndrome (MAS), or in more severe cases to CAR T-cell-related encephalopathy syndrome (CRES), which is characterized by confusion, delirium, and occasionally seizures and cerebral edema [8].

Binding of CARs to cognate antigens expressed on the surface of tumor cells induces T cell activation and subsequent release of various cytokines, including interleukin-2 (IL-2), interferon-γ (IFN-γ), IL-6, and granulocyte macrophage-colony stimulating factor (GM-CSF). The cytokines activate bystander immune cells, such as monocytes and macrophages, which secrete IL-6, IL-8, IL-10, macrophage inflammatory protein-1 alpha (MIP-1α), monocyte chemotactic protein-1 (MCP-1), and soluble IL-6 receptor (sIL-6R) [7, 9]. In CRS, extensive reciprocal signaling between T cells and macrophages occurs; hence, the discrimination of T cell overactivation from abnormal macrophage activation is challenging. Patients with severe CRS require intensive medical care with vasopressors, mechanical ventilation, antiepileptics, and antipyretics. The cytokine profile of patients undergoing CD19 CAR T-cell therapy has been associated with the severity of CRS; higher levels of IFN-γ, IL-6, IL-8, sIL-2Rα, sgp130, sIL-6R, MCP-1, MIP-1α, MIP-1β, and GM-CSF have been reported in patients with grade 4–5 CRS [9].

Although the administration of steroids can alleviate fever and other CRS-associated clinical symptoms in patients with CRS, steroids suppresses CAR T-cell expansion and persistence [10]. Moreover, the administration of alternative immune-suppressive agents, such as FK506 or cyclosporine, is not recommended, as their strong T cell-inhibitory effects impair the efficacy of CAR T-cell therapy and increases the risk of infectious disease [8]. Mouse studies conducted by Giavridis *et al*. [11] and Norelli *et al*. [12] revealed that IL-1 secretion by macrophages plays a pivotal role in CRS, suggesting the potential clinical benefit of the IL-1R antagonist anakinra. However, there is currently not enough evidence from clinical trials supporting the clinical benefit of anakinra in CRS patients. Tocilizumab is a humanized monoclonal antibody (mAb) against IL-6R. Patients who respond to tocilizumab recover from CRS without significant loss of CAR T-cell function; however, a significant portion of patients exhibit resistance to the effects of tocilizumab [7]. Furthermore, the effect of tocilizumab against severe CRS-associated neurotoxicity is extremely low, likely due to its limited ability to penetrate the blood-brain barrier [13]. Moreover, prophylactic use of tocilizumab did not prevent the development of neurotoxicity [14]. Therefore, IL-6/IL-6R blockade alone would be insufficient in treating severe CRS in patients undergoing CAR T-cell therapy.

We previously demonstrated that the investigational drug TO-207, formerly known as JTE-607, preferentially inhibits abnormal activation of macrophages without attenuating T-cell function [15–18]. TO-207 is an mRNA 3’-end processing antagonist that can inhibit the secretion of multiple cytokines [18]. Additionally, TO-207 treatment inhibited the *in vitro* production of IL-6, IL-8, tumor necrosis factor-alpha (TNF-α), IL-1β, IL-10, IL-1Rα, and GM-CSF in lipopolysaccharide (LPS)-stimulated peripheral blood mononuclear cells [15]. Importantly, although TO-207 treatment strongly suppressed cytokine secretion in monocytes [15, 16], it had no impact on cytokine production in human T cells *in vitro*.

In the current study, we developed an *in vitro* co-culture model that accurately recapitulates CAR T-related CRS, in which activated CAR T cells released IFN-γ, activating monocytes and cytokine release such as TNF-α, MIP-1α, M-CSF, IL-6, MCP-1, IL-1β, and IL-8. We report that a novel multi-cytokine inhibitor TO-207 specifically inhibits pro-inflammatory cytokines from monocytes, such as IL-6, IL-1β, MCP-1, IL-18, IL-8, and GM-CSF, without attenuating cytotoxicity by CAR T cells. Since the cytotoxicity is largely dependent on CAR T cells, selective inhibition of monocyte-derived cytokines by TO-207 would be an ideal treatment for CAR T–related CRS.

## Materials and methods

### Reagents

Prednisolone (PSL) was purchased from Fujifilm Wako (Osaka, Japan). TO-207 was purchased from Tocris Bioscience (Bristol, UK), and tocilizumab and anakinra were purchased from Absolute Antibody (Oxford, UK). LPS from E. coli 055: B55 and ATP were purchased from Sigma (St. Louis, MO, USA). Monensin solution (1000x) was purchased from BioLegend (San Diego, CA, USA).

### Cells

NALM-6 and K562 cells were obtained from the American Type Culture Collection. The cells were cultured in RPMI1640 medium (Fujifilm Wako) supplemented with 10% fetal bovine serum (FBS; Sigma) and 1% penicillin-streptomycin (Fujifilm Wako). Peripheral blood mononuclear cells (PBMCs) were harvested from healthy volunteers who gave written informed consent prior to collection. All relevant study-related protocols were approved by the institutional review boards of the Institute of Medical Science, the University of Tokyo (approval number 29–67). CD14^+^ and CD8^+^ cells in PBMCs were purified by magnetic sorting using human CD14 and CD8 microbeads (Miltenyi, Bergisch Gladbach, Germany).

### Antibodies

Anti-human CD3 mAb (Lymactin-T, 6001T01) was purchased from Cell Science & Technology (Sendai, Japan). Anti-human CD28 mAb (16-0289-85) was purchased from Thermo Fisher Scientific (Waltham, MA, USA). Flow cytometry was performed using allophycocyanin (APC)-conjugated IgG_1_ (BioLegend), APC–anti-human CD19 antibody (BioLegend), APC–anti human CD107a (BioLegend), APC–Cy7–anti-human CD3 antibody (BioLegend), V500–anti-human CD4 antibody (BD Biosciences, San Jose, CA, USA), and PerCP–Cy5.5–anti-human CD8 antibody (TONBO Biosciences, San Diego, CA, USA).

### Construction of lentiviral vectors

Anti-CD19 CAR (FMC63-28z) was constructed using the anti-CD19 scFv (FMC63); the hinge, transmembrane, and cytoplasmic domains of CD28; and the cytoplasmic domain of CD3-ζ based on the method by Kochenderfer *et al*. [19] The sequence was obtained from GenBank (HM852952), and codons were optimized using an IDT codon optimizing tool (http://sg.igtdna.com/CodonOpt) to avoid *Bam*HI sites within the open reading frame (for more detailed information, please refer to **S1 Text**). The optimized FMC63-28z sequence was chemically synthesized (FASMAC, Kanagawa, Japan) and amplified by polymerase chain reaction (PCR) using the following primer set:

5’- cgctaccggtctcgagaattcgccgccaccATGCTTCTC-3’ and

5’-gaagttcgtggctccggatccGCGAGGGGGCAG-3’.

The PCR product was inserted into the lentiviral backbone plasmid CSII-EF-MCS-2A-EGFP using the *Eco*RI and *Bam*HI sites, resulting in CSII-EF-FMC63-28z-2A-EGFP. To generate the CD19 expressing vector, the open reading frame of human CD19 was purchased from GenScript (Piscataway, NJ, USA) and amplified by PCR using the following primer set:

5’-ccggtctcgagaattcgccaccATGCCACCTCCTCGCCTC-3’ and

5’-cgatgttaactctagatTCACCTGGTGCTCCAGGTGC-3’.

The PCR product was inserted into the lentiviral backbone plasmid CSII-EF-MCS using the *Eco*RI and *Xba*I sites, resulting in CSII-EF-hCD19. To generate the plasmid expressing firefly luciferase (fLuc), fLuc cDNA was amplified by PCR with the following primer set:

5’-gaattcgccaccATGGAAGATGCCAA-3’ and 5’-ggatccCACGGCGATCTTGCCGCC-3’.

The pmirGLO plasmid (Promega, Madison, WI, USA) was used as a template. The PCR product was cloned into CSII-EF-MCS-2A-EGFP using the *Eco*RI and *Bam*HI sites, resulting in CSII-EF-fLuc-2A-EGFP. Lentiviral vector particles were produced by the co-transfection of Lenti-X293T cells (Clontech, Mountain View, CA, USA) with a transfer plasmid, and packaging plasmids pMDLg/p.RRE, pRSV-rev, and pMD.G.

### *In vitro* T-cell expansion

Suspension culture flasks (Sumitomo Bakelite, Tokyo, Japan) were pre-coated with 5 μg/ml anti-CD3 mAb and 5 μg/ml anti-CD28 mAb by incubation at room temperature for 45 min. PBMCs (1 × 10^5^–1 × 10^6^/ml) were suspended in AlyS505N-7 medium (Cell Science & Technology) containing 700 IU/ml IL-2, 5% FBS, and 1% penicillin-streptomycin-amphotericin B (Fujifilm Wako). The cells were then seeded into the flacks pre-coated with anti-CD3 and anti-CD28 mAbs. For short-term stimulation of CD8^+^ T cells derived from PBMCs, Dynabead human T-activator CD3/CD28 (Thermo Fisher Scientific) was used.

### Generation of CAR T cells and target cells via lentiviral transduction

PBMCs were cultured in flasks pre-coated with anti-CD3 and anti-CD28 mAbs for 2 days, and 2 × 10^6^ cells were transduced with the FMC63-28z-2A-EGFP lentiviral vector at a multiplicity of infection of five. Two days after transduction, CAR T cells (CD3^+^, EGFP^+^) were harvested by fluorescence-activated cell sorting and expanded for an additional 10 days. CD4^+^ and CD8^+^ CAR T cells were separated by magnetic sorting using human CD8 microbeads (Miltenyi). NALM-6 and K562 cells were transduced with lentiviral vectors that expressed fLuc-2A-EGFP or CD19 at a multiplicity of infection of five. Three days after transduction, the cells were purified by fluorescence-activated cell sorting.

### Luciferase assay

The luciferase assay was performed using the Picagene LT7.5 luminescence kit (Fujifilm Wako) as per the manufacturer’s protocol.

### Cytokine assay

Cytokines in the co-culture consisting of K562/CD19 cells, CAR T cells, and CD14^+^ cells were determined by cytokine bead array using the HQPlex premix multiple kit (Bay Bioscience, Kobe, Japan). Cytokines in Dynabead-stimulated T cells or LPS-stimulated CD14^+^ cells were determined by enzyme-linked immunosorbent assay (human DuoSet; R&D systems, Minneapolis, MN, USA).

### Degranulation assay

K562/CD19 cells (5 × 10^5^) and CAR T cells (5 × 10^5^) were suspended in 500 μl T-cell expansion medium (ALys505N-7 containing 5% FBS and 1% penicillin-streptomycin-amphotericin B). Cells were cultured in a 24-well plate in the absence or presence of PSL (0–100 μM) or TO-207 (0–1000 nM). Following 68 h of co-culture, monensin (final concentration = 2 μM) and APC–anti-human CD107a or APC-IgG_1_ (10 μL) were added. Cells were incubated for an additional 4 h. The surface expression of CD107a in the CAR T cell fraction (EGFP^+^) was determined by flow cytometry.

## Statistical analysis

The linear dose-response relationship was assessed using log-transformed dose values (to the base 10) in a mixed model, in which the zero dose was replaced by the log (minimal dose) - 1. Differences in dose- or time-dependent changes between two groups were assessed using a two-factor linear mixed-effect model with interaction terms. In this model, the dose variable was entered as a continuous variable after log-transformation, and the time variable was entered as a categorical variable because the correlation with the outcome was not linear.

## Results

### Establishment of an *in vitro* CRS model using a K562/CD19 cell, 19-28z CAR T cell, and CD14^+^ peripheral blood mononuclear cell (PBMC) co-culture system

**Fig 1:**
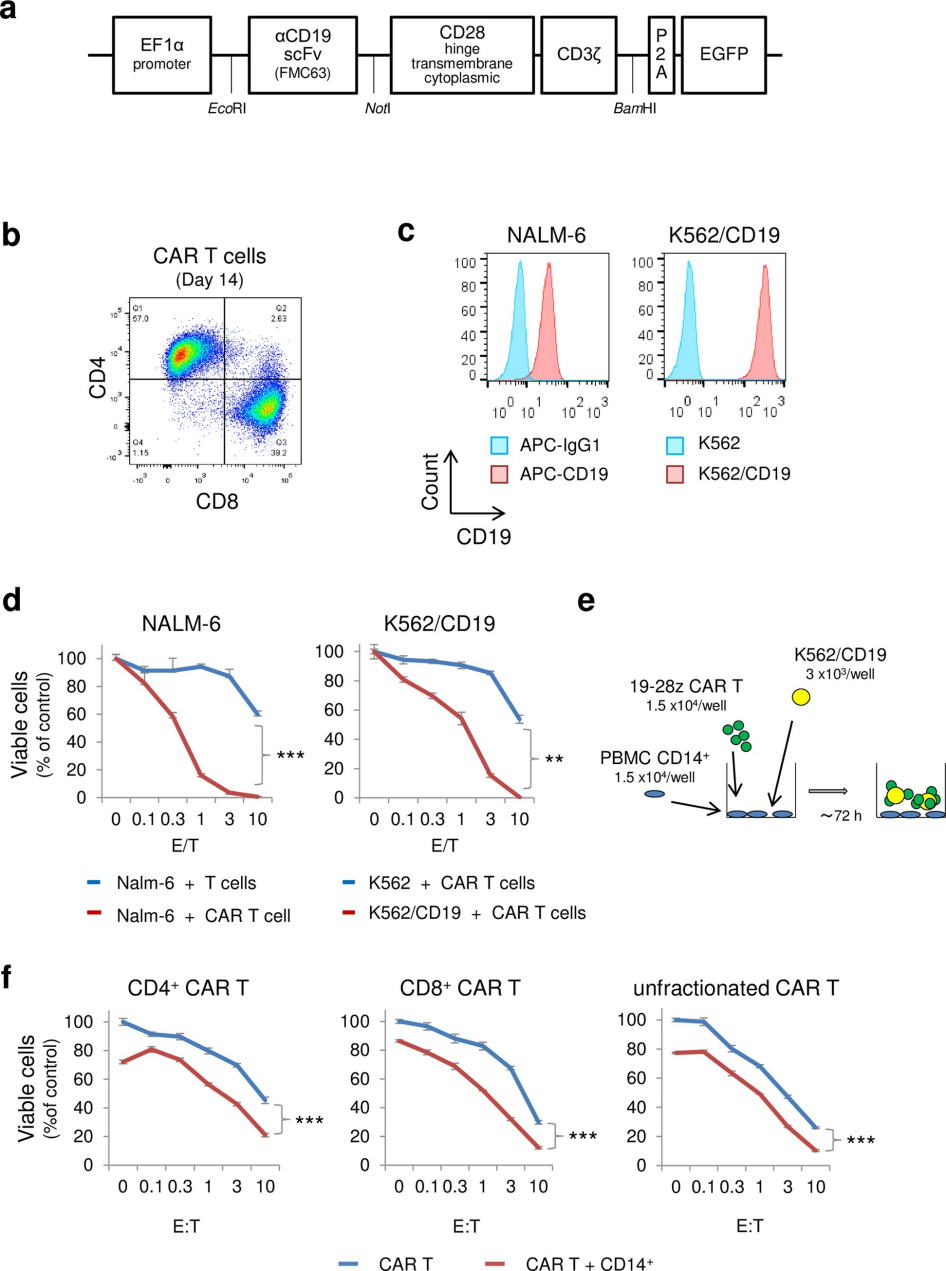
Establishment of an in vitro CRS model using a K562/CD19 cell, 19-28z CAR T-cell, and CD14^+^ peripheral blood mononuclear cell (PBMC) co-culture system. A) Construction of the 19-28z chimeric antigen receptor (CAR) using the anti-CD19 scFv (clone FMC63), the hinge, transmembrane, and cytoplasmic domains of CD28, and the cytoplasmic domain of the CD3-ζ chain. B) CD4 and CD8 expression in CAR T cells. Following 14 days of *in vitro* expansion, the percentages of CD4^+^ and CD8^+^ CAR T cells were determined by flow cytometry. C) CD19 expression in target cells (NALM-6 cells, and CD19-transduced K562 cells), determined by flow cytometry. D) Cytotoxicity of CAR T cells on NALM-6 cells and K562/CD19 cells. Target cells (NALM-6/fLucEGFP, K562/fLucEGFP, and K562/CD19/fLucEGFP) were co-cultured with CAR T cells (E/T = 0–10), and the numbers of viable cells were determined using the luciferase assay. As a control, peripheral blood T cells were cultured for 14 days *in vitro*. E) CRS co-culture model. K562/CD19 cells (3 × 10^3^), 19-28z CAR T cells (1.5 × 10^4^), and CD14^+^ PBMCs (1.5 × 10^4^) were co-cultured in a 96-well plate for up to 72 h. F) Cytotoxicity of CD4^+^, CD8^+^, and unfractionated CAR T cells. K562/CD19/fLucEGFP cells (3 × 10^3^) and CD4^+^, CD8^+^, or unfractionated 19-28z CAR T cells (0–3 × 10^4^) were co-cultured in a 96-well plate in the absence or presence of CD14^+^ PBMCs (1.5 × 10^4^). After 72 h of co-culture, viable cells were quantified by the luciferase assay. Values were normalized to the control well containing only K562/CD19/fLucEGFP. The error bars represent standard deviations (SDs) from three independent experiments. Difference in dose-dependent changes between two groups were assessed using a two-factor linear mixed-effect model with interaction terms. In this model, the dose variable was entered as a continuous variable after log-transformation (to the base 10), in which the zero dose was replaced by the log (minimal dose) - 1. (*p < 0.05, **p < 0.01. ***p < 0.001).

A lentiviral vector that expresses anti-CD19 CAR was generated using anti-CD19 scFv (derived from clone FMC63), CD28 (hinge, transmembrane, and cytoplasmic domain), and CD3ζ chain (**Fig** 1A). Following lentiviral transduction, the 19-28z CAR T cells were sorted using enhanced green fluorescence protein (EGFP) expression, and expanded *ex vivo* for 14 days by stimulating with anti-CD3 antibody, anti-CD28 antibody, and human IL-2. The percentage of CD4^+^ cells and CD8^+^ cells on day 14 were 57.0% and 39.2%, respectively (**Fig** 1B). For the target cell, NALM-6 (derived from B-acute lymphoblastic leukemia), and K562/CD19 cells (derived from chronic myeloid leukemia, lentivirally transduced with CD19) were chosen, and their CD19 expressions were confirmed by flow cytometry (**Fig** 1C). A clear dose-dependent cytotoxic effect of 19-28z CAR T cells was validated by a 72h co-culture, in which target cells (NALM-6, K562/CD19) and CAR T cells were mixed at the effector to target ratio (E/T) of 0~10 (**Fig** 1D). Since K562 cells lack human leukocyte antigen (HLA) class I and II [20] and therefore avoid alloreactivity to observe CAR T specific cytotoxicity, we chose K562/CD19 cells as the target in this study. As cytokines are released not only by CAR T cells, but also by monocytes and their myeloid derivatives, we established a co-culture system that models CRS using K562/CD19 cells, 19-28z CAR T cells, and CD14^+^ PBMCs (**Fig** 1E). CD4^+^ CAR T cells and CD8^+^ CAR T cells were isolated by magnetic cell sorting; both cell types exhibited dose-dependent cytotoxicity when co-cultured with K562/CD19 cells. The addition of CD14^+^ cells to the co-culture system significantly enhanced cytotoxicity (*p* < 0.001); however, the cytotoxicity was largely mediated by CAR T cells, and to a lesser extent by PBMCs (**Fig** 1F).

### CRS-related, pro-inflammatory cytokine production was dependent on CD14^+^ monocytes

**Fig 2:**
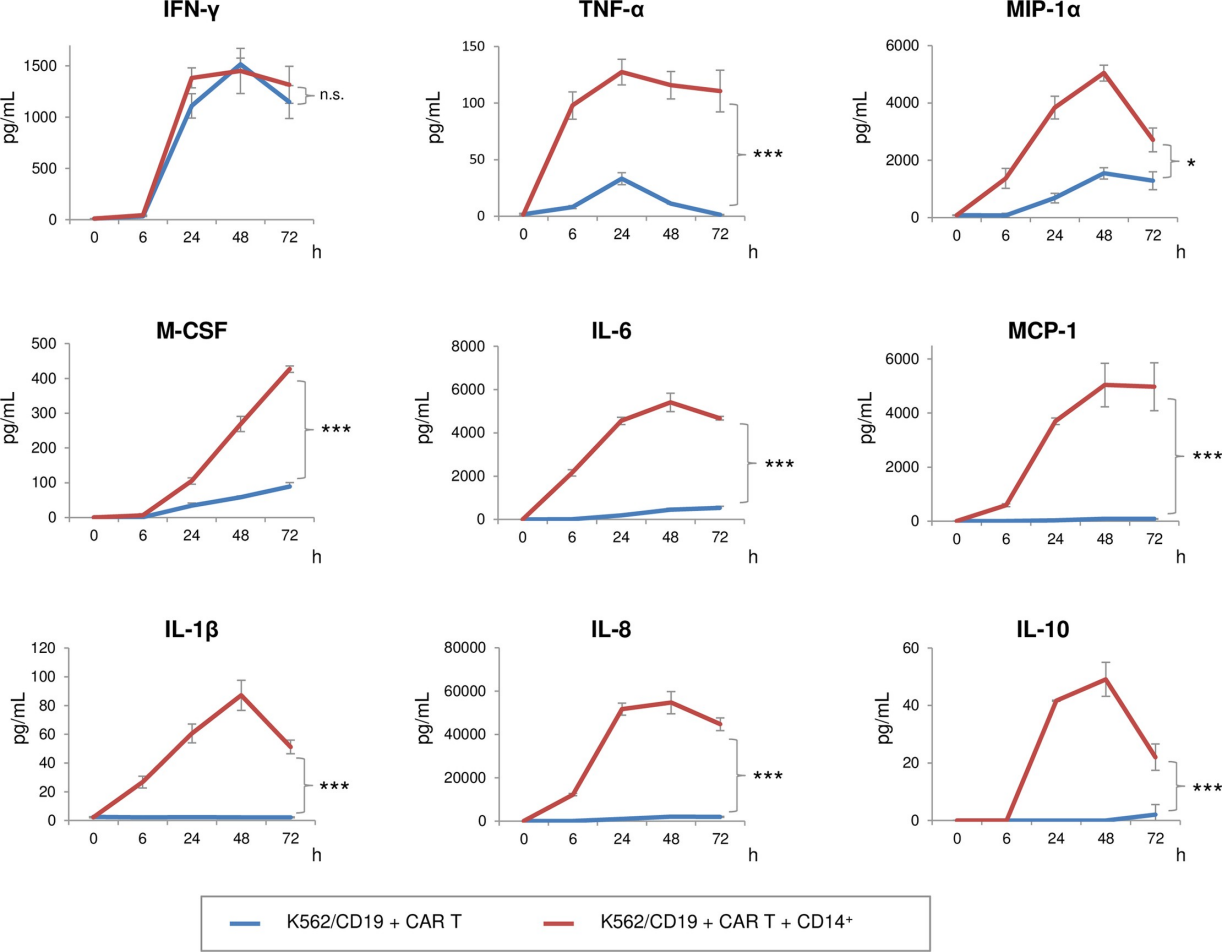
CRS-related, pro-inflammatory cytokine production was dependent on CD14^+^monocytes. Time course of cytokine production. Supernatants in the absence (blue line) or presence (red line) of CD14^+^ PBMCs were recovered, and cytokines were quantitated using a cytokine bead array. The error bars represent SDs from three independent experiments. Difference in time-dependent changes between two groups were assessed using a two-factor linear mixed-effect model with interaction terms, in which the time variable was entered as a categorical variable because the correlation with the outcome was not linear. *P <* 0.05 was considered statistically significant (*p < 0.05, **p < 0.01. ***p < 0.001). n.s.: not significant.

To observe a precise pattern of cytokine production by CAR T cells and monocytes, K562/CD19 cells and CAR T cells were co-cultured in the presence or absence of CD14^+^ monocytes. Pattern and time-course of cytokine productions were also determined (**Fig** 2). CAR T cells predominantly produced IFN-γ, relative to CD14^+^ monocytes. Both CAR T cells and monocytes produced TNF-α, MIP-1α, macrophage colony-stimulating factor (M-CSF), and IL-6, but monocytes were the major source of these cytokines. MCP-1, IL-1β, IL-8, and IL-10 were produced exclusively by monocytes (**Fig** 2). These results suggested that CRS-related cytokine production depends largely on CD14^+^ monocytes.

### Pharmacological effects of TO-207 and prednisolone on cytokine production in activated T cells and CAR T cells

**Fig 3:**
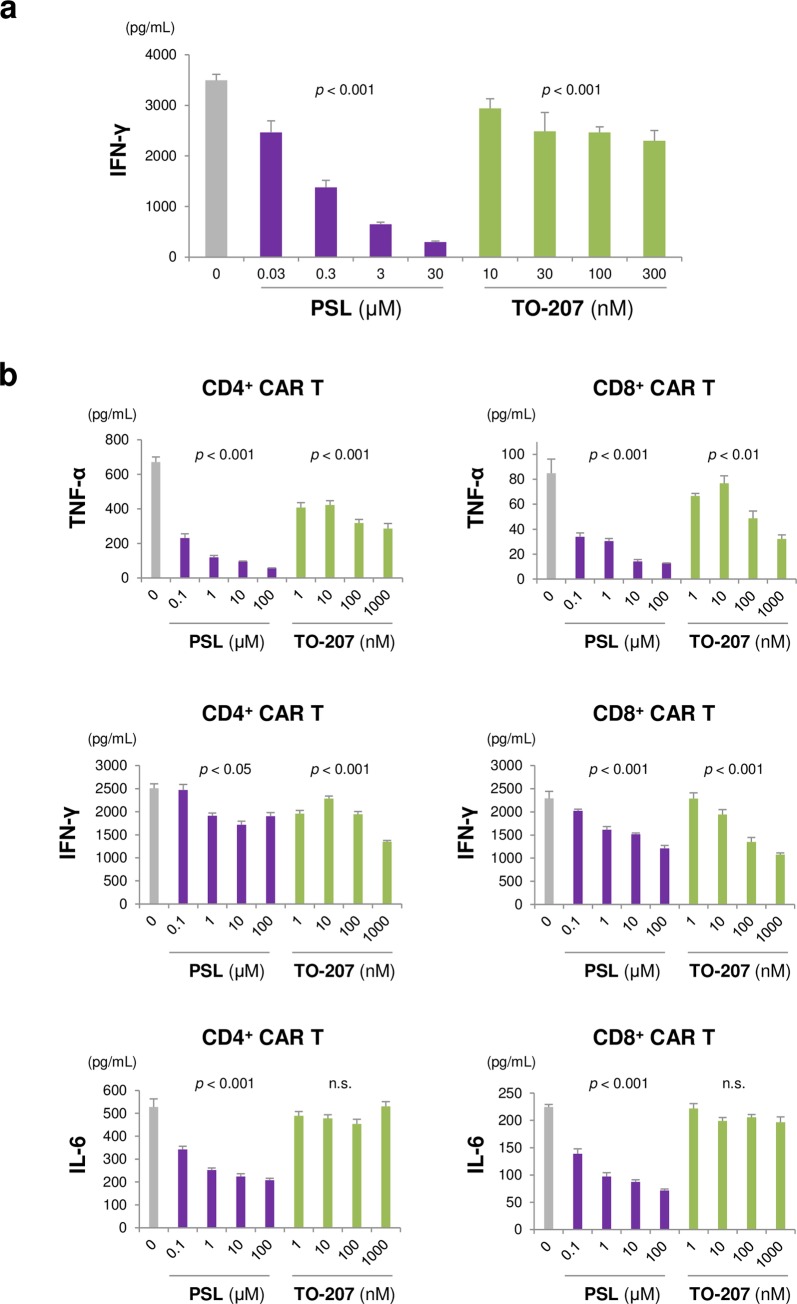
Pharmacological effects of TO-207 and prednisolone on cytokine production in activated T cells and CAR T cells. A) Effects of PSL and TO-207 on activated CD8^+^ T cells. Peripheral blood CD8^+^ T cells (2 × 10^4^/well) were activated with anti-CD3 and anti-CD28 mAbs (Dynabeads human T-activators CD3 and CD28), and treated with PSL or TO-207. Supernatants were recovered after 72 h. The error bars represent standard errors (SEs) from three independent experiments. B) Effects of PSL and TO-207 on CAR T cell cytokine production. K562/CD19 cells (1 × 10^4^) were co-cultured with CD4^+^ or CD8^+^ CAR T cells (5 × 10^4^). The cells were treated with different concentrations of PSL and TO-207. Supernatants were recovered 24 h later. The error bars represent SEs from three independent experiments. The linear dose-response relationship was assessed using log-transformed dose values (to the base 10) in a mixed model, in which the zero dose was replaced by the log (minimal dose) - 1. *P <* 0.05 was considered statistically significant. n.s.: not significant.

The novel cytokine inhibitor TO-207 has been previously reported to inhibit cytokine production in a myeloid lineage-specific manner [16]. Thus, we hypothesized that TO-207 could mitigate CRS without impacting CAR T cell-mediated cytotoxicity. To validate this hypothesis, we treated CD3/CD28-stimulated CD8^+^ T cells with prednisolone (PSL) and TO-207. We found that both drugs had dose-dependent effects on cytokine production; however, the effects exerted by PSL were more profound than those exerted by TO-207 (**Fig** 3A). To confirm whether this result extended to CAR T cells, we co-cultured CD4^+^ or CD8^+^ CAR T cells with K562/CD19 cells in the presence or absence of PSL or TO-207, and determined the levels of secreted TNF-α, IFN-γ, and IL-6. PSL significantly suppressed TNF-α production, even at a very low concentration (0.1 μM; **Fig** 3B), and modestly suppressed IFN-γ and IL-6 secretion (**Fig** 3B). In contrast, TO-207 had modest effects on TNF-α and IFN-γ production, and no effects on IL-6 secretion (**Fig** 3B). These results suggest that TO-207 has a weak, if any, effect on cytokine secretion in CAR T cells.

### Suppression of monocyte-derived, pro-inflammatory cytokine secretion by TO-207 and IL-6/IL-1 pathway-specific suppression by tocilizumab/anakinra

**Fig 4:**
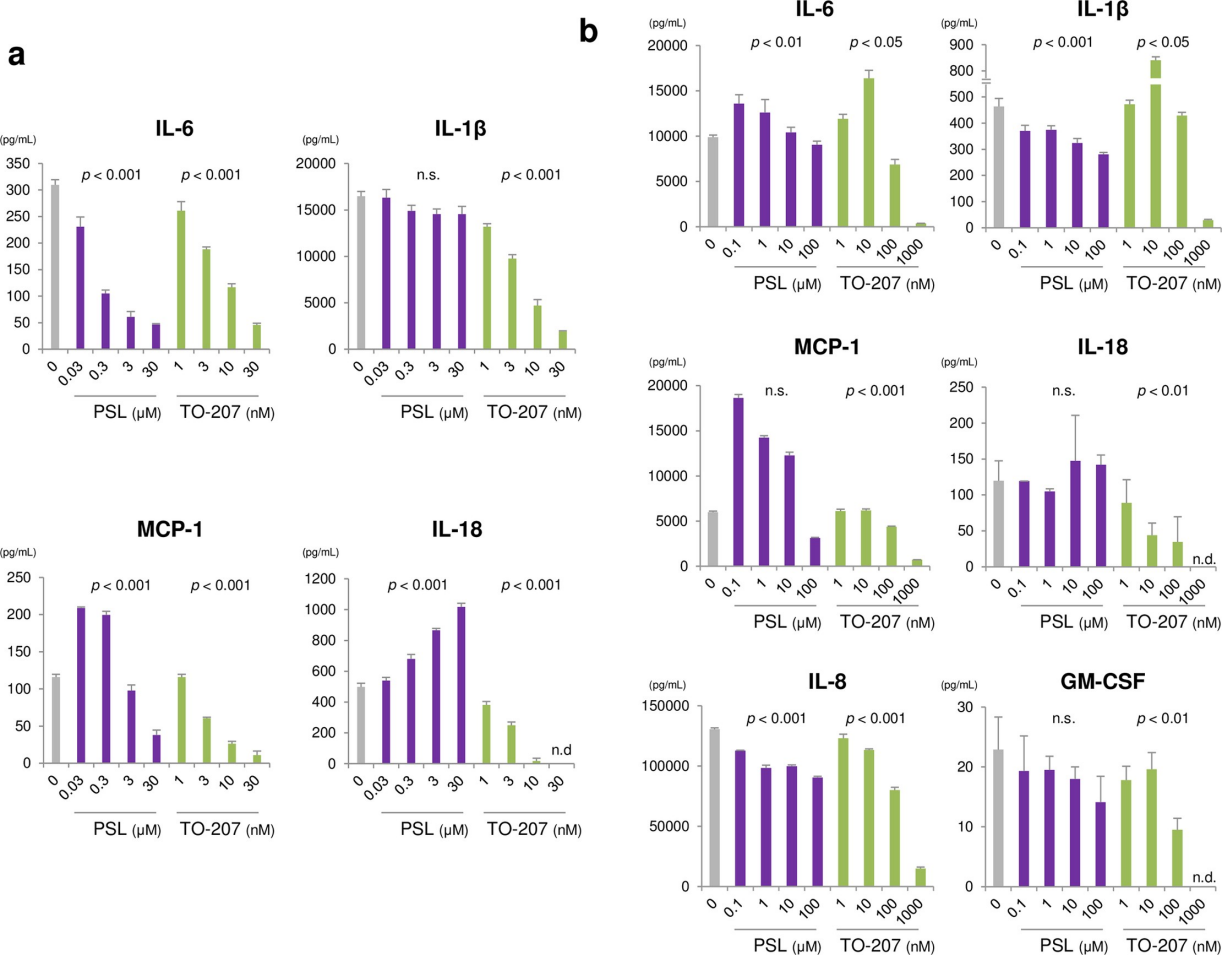
Suppression of monocyte-derived, pro-inflammatory cytokine secretion by TO-207 and IL-6/IL-1 pathway–specific suppression by tocilizumab/anakinra. A) Effects on cytokine production by LPS- and ATP-stimulated monocytes. Peripheral blood mononuclear CD14^+^ cells (2 × 10^5^) were seeded in a 96-well plate with culture medium containing PSL or TO-207. The cells were stimulated with 100 ng/mL LPS for 3 h, and then with 5 mM ATP for 2 h. Supernatants were recovered, and cytokine levels were determined. The error bars represent SEs from three independent experiments. B) Wide-ranging suppression of monocyte-derived pro-inflammatory cytokines by TO-207. K562/CD19 cells (3 × 10^3^), CAR T cells (1.5 × 10^4^), and CD14^+^ cells (1.5 × 10^4^) were co-cultured in a 96-well plate in the absence or presence of PSL or TO-207. After 72 h of co-culture, the supernatants were recovered, and cytokine levels were determined. The error bars represent SEs from three independent experiments. The linear dose-response relationship was assessed using log-transformed dose values (to the base 10) in a mixed model, in which the zero dose was replaced by the log (minimal dose) - 1. *P* < 0.05 was considered statistically significant. n.s.: not significant. n.d.: not detected.

To examine the effects of PSL and TO-207 on cytokines in monocytes, we used an LPS- and adenosine 5’-triphosphate (ATP)-mediated monocyte stimulation approach. In contrast to what we observed in T cells, both PSL and TO-207 suppressed the secretion of monocyte-derived cytokines, including IL-6 and MCP-1, in a dose-dependent manner. However, TO-207 was more potent than PSL in suppressing the secretion of monocyte-derived cytokines (**Fig** 4A). Additionally, TO-207 suppressed the production of IL-1β, IL-18, and osteopontin in a dose-dependent manner (**Fig** 4A, **S1 Fig**). To further examine whether TO-207 and other therapeutic drugs could suppress cytokine production in monocytes stimulated by CAR T cells, we co-cultured K562/CD19 cells, CAR T cells, and CD14^+^ cells in the presence of PSL, TO-207, tocilizumab, or the recombinant IL-1R antagonist anakinra. PSL (100 μM) suppressed IL-6, IL-1β, and IL-8 production. Moreover, MCP-1 was upregulated in response to a very low PSL concentration (0.1 μM), suggesting a concentration-dependent regulation of anti- and pro-inflammatory cytokines (**Fig** 4B). TO-207 suppressed the secretion of all monocyte-derived cytokines tested, including IL-6, IL-1β, MCP-1, IL-18, IL-8, and GM-CSF. Nevertheless, very low concentrations of TO-207 (~10 nM) upregulated IL-6 and IL-1β (**Fig** 4B). Tocilizumab did not suppress cytokine levels. Contrary to our expectations, tocilizumab significantly increased MCP-1 secretion (**S2 Fig**). Anakinra significantly increased IL-6 and IL-1β production, suggesting the activation of a negative feedback loop (**S3 Fig**). These results suggest that, unlike other immune-suppressive drugs, TO-207 suppresses the secretion of a wide range of monocyte-derived CRS-related cytokines.

### Advantageous pharmacological effects of TO-207 from steroid on cytotoxic effect of activated CAR T cells in the co-culture model

**Fig 5:**
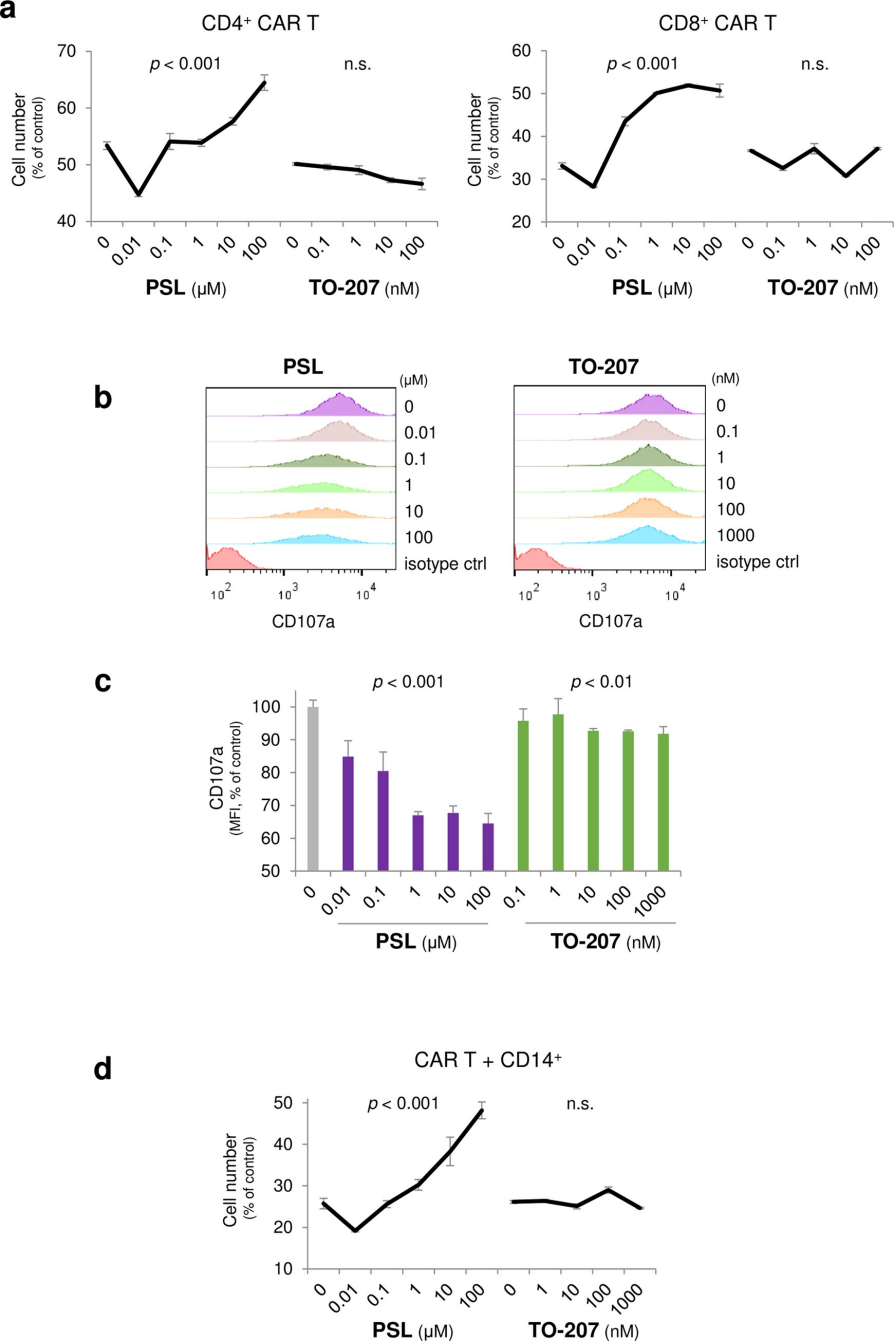
Advantageous pharmacological effects of TO-207 from steroid on cytotoxic effect of activated CAR T cells in the co-culture model. A) Effects of PSL and TO-207 on the cytotoxicity of CAR T cells. K562/CD19/fLucEGFP cells (3 × 10^3^) were co-cultured with CD4^+^ or CD8^+^ CAR T cells (1.5 × 10^4^), and treated with different concentrations of PSL and TO-207. Viable target cells were quantified using the luciferase assay after 72 h of co-culture. Values were normalized to viable K562/CD19/fLucEGFP cells cultured alone. The error bars represent SDs from three independent experiments. B) Effects of PSL and TO-207 on CAR T cell degranulation. K562/CD19 cells (5 × 10^5^) and CAR T cells (5 × 10^5^) were co-cultured in 500 μl T-cell expansion medium in the absence or presence of PSL or TO-207. Following 68 h of co-culture, monensin (2 μM) and APC–anti-human CD107a antibody were added. Cells were incubated for an additional 4 h, and membrane expression of CD107a was determined by flow cytometry. Representative data of four independent experiments are shown. C) Mean fluorescence intensity (MFI) of samples in Fig 5B. The error bars represent SEs from four independent experiments. D) Sustained cytotoxicity of CAR T cells following TO-207 treatment. K562/CD19/fLucEGFP cells (1 × 10^4^), CAR T cells (5 × 10^4^), and CD14^+^ cells (5 × 10^4^) were co-cultured in the absence or presence of PSL or TO-207 for 72 h, and viable target cells were quantified by the luciferase assay. Values were normalized to the well containing K562/CD19/fLucEGFP cells alone. The error bars represent SDs from three independent experiments. The linear dose-response relationship was assessed using log-transformed dose values (to the base 10) in a mixed model, in which the zero dose was replaced by the log (minimal dose) - 1. *P <* 0.05 was considered statistically significant. n.s.: not significant.

To determine whether TO-207 affects the cytotoxic effect of CAR T cells, we observed the direct effect on CAR T cells of TO-207 in the absence of CD14^+^ cells. Although a very low concentration of PSL (0.01 μM) slightly enhanced cytotoxicity of CD4^+^ and CD8^+^ CAR T cells, the effect may be presumably due to inhibition of anti-inflammatory cytokines from T cells, higher concentrations of PSL (1~100 μM) clearly deteriorated therapeutic effect of CD4^+^ CAR T cells and CD8^+^ CAR T cells toward K562/CD19 cells (**Fig** 5A). In contrast, TO-207 did not have any effect on cytotoxicity of CD4^+^ CAR T and CD8^+^ CAR T cells (**Fig** 5A). To further confirm whether PSL and TO-207 affect cytotoxic function of CAR T cells, we co-cultured CD8^+^ CAR T cells and K562/CD19 cells (E/T = 1) in the presence or absence of either PSL or TO-207. T-cell degranulation was evaluated by flow cytometry detecting cell surface CD107a. Treatment with PSL significantly inhibited CD8^+^ CAR T cell degranulation in a dose-dependent manner (**Fig** 5B and 5C), whereas TO-207 minimally affected degranulation of CD8^+^ CAR T cells (**Fig** 5B and 5C). To determine whether addition of CD14^+^ monocytes changes the effect of PSL and TO-207 on cytotoxicity, K562/CD19 cells, CAR T cells, and CD14^+^ cells were co-cultured. PSL significantly attenuated cytotoxicity, whereas TO-207 had no effect on cytotoxicity (**Fig** 5D). These results suggested that PSL inhibited the cytotoxicity of CAR T cells, which hinders its usefulness during concomitant CAR T therapy, but TO-207 maintains the cytotoxic effects toward tumor cells.

## Discussion

**Fig 6:**
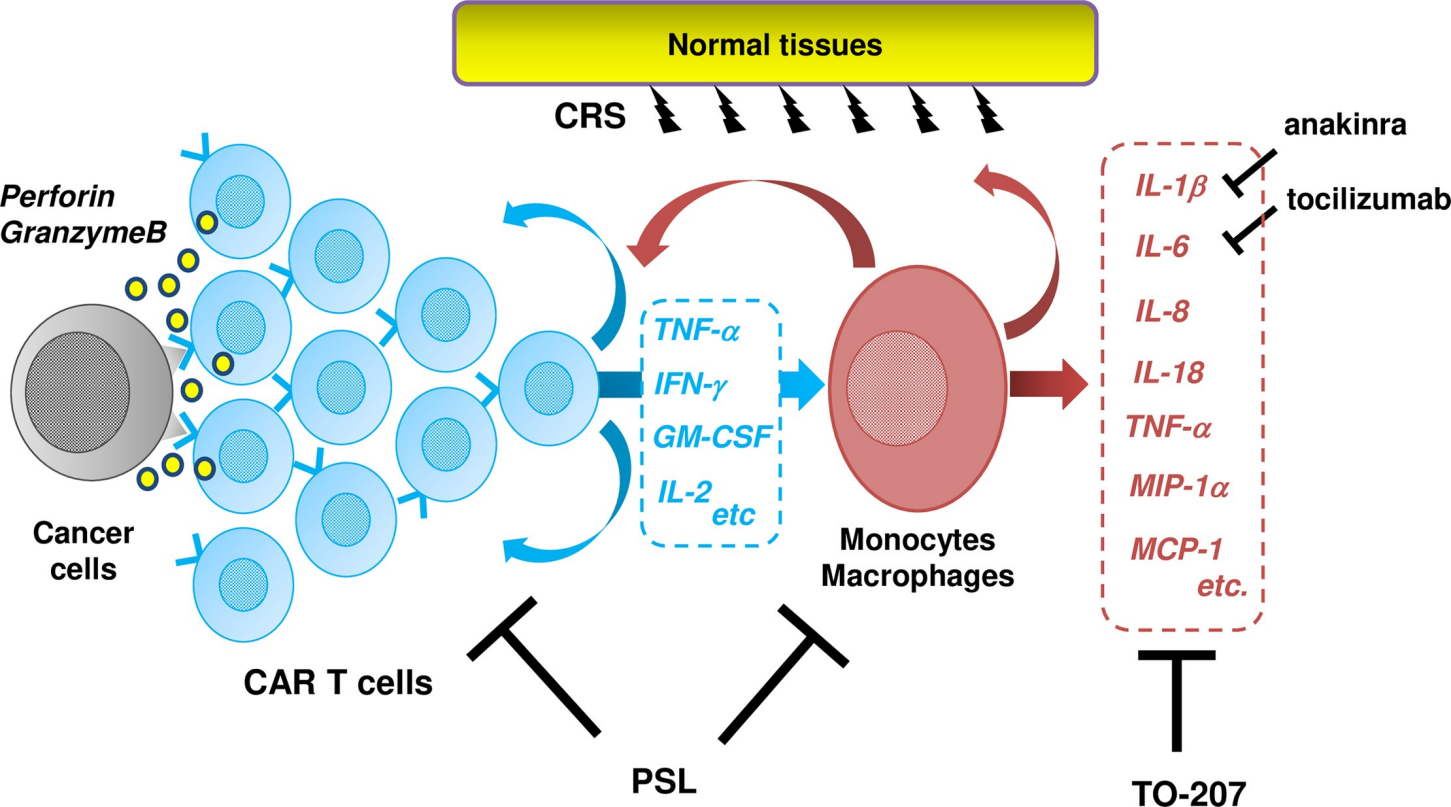
Targeted effects of TO-207 on monokine production. Interactions among cancer cells, CAR T cells, and monocytes and macrophages in CRS, and therapeutic actions of PSL, TO-207, anakinra, and tocilizumab. PSL suppressed cytokine production derived from T cells and monocyte and macrophages while attenuating the cytotoxic effects of CAR T cells, whereas TO-207 suppressed monokine production without disturbing the cytotoxic effects of CAR T cells. Anakinra and tocilizumab did not exert suppressive effects on cytokines other than their specific targets.

CRS is a severe, life-threatening adverse event, requiring novel treatment with better efficacy. However, there is scarcely an *in vitro* CRS model, which makes it difficult to develop novel agents in a high-throughput manner. Teachey *et al*. [9] compared cytokine profiles in patients who underwent CD19 CAR T therapy, and reported higher levels of IFN-γ, IL-6, IL-8, sIL-2Rα, sgp130, sIL-6R, MCP-1, MIP-1α, MIP-1β, and GM-CSF were associated with grade 4–5 CRS. Giavridis *et al*. [11] developed a mouse CRS model using a xenograft of human CD19^+^ Raji tumor and 19-28z CAR T cells into SCID-beige mice. Their RNA-sequencing analysis revealed significant up-regulation of IL-1Rs and IL-1R antagonists in macrophages suggesting a response to hyperactive IL-1 signal. Treatment with anakinra significantly prolonged overall survival of CRS mice.[11] Norelli *et al*. [12] also developed a CRS mouse model using transplantation of leukemia cells and CAR T cells into humanized SGM3 mice and reproduced a severe CRS and lethal neurotoxicity. RNA-sequencing revealed monocytes were the major source of IL-1 and IL-6 during CRS. Tocilizumab prevented CRS by IL-6R blockade, but failed to protect mice from delayed lethal neurotoxicity while anakinra abolished both CRS and neurotoxicity in the models. Our present co-culture model using K562/CD19 cells, 19-28z CAR T cells, and CD14^+^ monocytes displayed a significant up-regulation of IFN-γ, TNF-α, MIP-1α, M-CSF, IL-6, MCP-1, IL-1β, IL-8, and IL-10. The pattern of cytokine production was very similar to those reported in clinical studies with severe CRS patients and recently developed mouse CRS xenograft models. IFN-γ was produced exclusively by CAR T cells, but most of cytokines such as TNF-α, MIP-1α, M-CSF, IL-6, MCP-1, IL-1β, IL-8, and IL-10 were from CD14^+^ monocytes. Our model accurately recapitulated cytokine release profiles of CRS, and it could be a useful tool for drug screening. The killing effect was largely dependent on CAR T cells while cytokine production was dependent on monocytes, selective inhibition of pro-inflammatory cytokines from monocytes would be most suitable for CAR T-related CRS therapy. Tocilizumab is currently a treatment of choice for CRS, as it does not significantly impact CAR T-cell function [7]. However, several patients are resistant to therapeutic effects of tocilizumab. Moreover, patients with CRS-related neurotoxicity do not benefit from tocilizumab due to its poor penetration through the blood-brain barrier [14]. Hence, the development of novel therapeutic approaches for CRS remains an unmet clinical need. Using an *in vitro* CRS model, we found that TO-207 preferentially inhibited monocyte-derived inflammatory cytokine production, making it a promising candidate for clinical use in CRS patients (**Fig** 6).

TO-207 formerly known as JTE-607, an N-benzoyl-L-phenylalanine derivative compound, is a novel inhibitor of multiple cytokines [18]. TO-207 is thought to inhibit cytokine production by antagonizing mRNA 3’-end processing and maturation [18]. 3’-End processing requires multi-subunit complexes, including the cleavage and polyadenylation specificity factor (CPSF), cleavage stimulation factor (CstF), cleavage factor I, cleavage factor II, poly(A) polymerase, and scaffold proteins such as symplekin [21, 22]. CPSF binds to the consensus poly(A) signal AAUAAA and CstF binds to a GU-rich element at the 3’ end. CPSF carries out endonucleolytic cleavage about 21 nucleotides downstream of the poly(A) signal. Kakegawa *et al*.[18] showed that TO-207/JTE-607 exerts its effect by binding directly to CPSF3. LPS-stimulated monocytes upregulated the transcription of IL-8 mRNA, but the addition of TO-207 significantly inhibited the maturation of IL-8 pre-mRNA, resulting in a reduction of the IL-8 protein level.[18] TO-207 was shown to inhibit the *in vitro* production of IL-6, IL-8, TNF-α, IL-1β, IL-10, IL-1Ra and GM-CSF by LPS-stimulated PBMCs with a very low IC_50_ (between 2.4 and 11.0 nM) [15]. In addition, Tajima *et al*.[16] reported that TO-207 inhibited cytokine production and autocrine/paracrine-dependent proliferation of leukemia cell lines, and the effect was 5- to 100-fold more profound in myelo-/monocytic cells than in lymphoid cells. The precise mechanism underlying monocytic lineage-specific action is still unknown, but the quantity and components of the 3’-end processing machinery must be recognized. Shell *et al*.[23] reported that LPS-stimulated murine RAW254.7 macrophages upregulated CstF-64 expression, and forced overexpression of CstF-64 altered gene expression very similarly to LPS-stimulation.[23] Therefore, activated macrophages are likely to be more susceptible to agents that alter 3’-end processing. These findings could explain the monocytic lineage-dominant action of TO-207. Monocytes and macrophages are powerful modulators of immune responses in tumor microenvironments. Recent studies have revealed that chronic inflammatory circumstances trigger the generation of myeloid-derived suppressor cells (MDSCs) [24, 25]. GM-CSF, in combination with TNF-α, IL-6, and IL-1β induce generation, activation, expansion, and migration of MDSCs [24, 26–30]. Activated MDSCs suppress anti-tumor immunity by various mechanisms, including activation of inducible NO synthase and arginase-1 [24, 31] and induction of anti-inflammatory cytokines such as IL-10 [24, 32]. Most of the cytokines that induce and/or activate MDSCs can be targeted by TO-207, therefore, TO-207 may support anti-tumor immunity via the inhibition of MDSCs. Ross *et al*.[33] recently reported that TO-207/JTE-607 could bind to CPSF3, inhibiting the proliferation of acute myelogenous leukemia and Ewing’s sarcoma cell lines by suppressing the aberrant expression of downstream oncogenic pathways. These results suggest that TO-207 can suppress tumor progression through multiple pathways.

*In vivo* disease models have also shown the therapeutic and prophylactic effects of TO-207. These include a mouse model of LPS induced shock [15], a lethal acute lung injury model in endotoxemic mice following burn insult [34], a mouse septic shock model using cecal ligation and puncture [35], a mouse model of ischemia-reperfusion injury in the myocardium [36], and an acute leukemia model with U-937 cells [37]. In all these animal models, TO-207 ameliorated disease symptoms and prolonged survival of animals, and was associated with suppression of pro-inflammatory cytokines production. Future studies in mouse models are required to demonstrate the ability of TO-207 to attenuate CRS. However, its potential species-specific activity due to the two amino acid changes in the TO-207 binding domain of CPSF3 between human and mouse (M332/S334 in human CPSF3, I332/N334 in mouse CPSF3) makes mouse experiments challenging [33]. Murine cells are less sensitive to TO-207 by 400-fold compared with human cells [15]. Based on its pharmacological characteristics and preclinical toxicological evaluation in monkeys, TO-207 has been used in clinical trials, including in healthy volunteers undergoing endotoxin challenge and patients with systemic inflammatory response syndrome [17]. Continuous infusion of TO-207 at doses of 0.1 and 0.3 mg/kg/h largely suppressed the secretion of pro-inflammatory cytokines, including IL-1β, IL-6, IL-8, IL-10, and C-reactive protein, in endotoxin-challenged humans [17]. Thus, several lines of evidence exist to support the effectiveness of TO-207 in CRS *in vivo*.

In conclusion, we developed an *in vitro* co-culture model that accurately recapitulated CAR T-related CRS. Activated CAR T cells released IFN-γ, activating monocytes and cytokine release such as TNF-α, MIP-1α, M-CSF, IL-6, MCP-1, IL-1β, and IL-8. While CAR T cells play the major role in cytotoxic effect against tumor cells, excessive production of pro-inflammatory cytokines from monocytes and macrophages cause CRS. Steroids suppress the function of both CAR T and monocytes and attenuate cytotoxic effect toward tumor cells. Tocilizumab and anakinra specifically inhibit their target molecules but they cannot inhibit the other monocyte-derived cytokines. Our results suggested that some cytokines could even be up-regulated due to activation of alternative pathways. TO-207, a novel multi-cytokine inhibitor, specifically inhibits pro-inflammatory cytokines from monocytes, such as TNF-α, IL-6, IL-1β, MCP-1, IL-18, IL-8, and GM-CSF, without affecting cytokine production and cytotoxicity by CAR T cells. Since the cytotoxicity is largely dependent on CAR T cells, selective inhibition of monocyte-derived cytokines by TO-207 would be an ideal treatment for CAR T–related CRS. These results encourage us to consider use of TO-207 in a clinical study to ameliorate CRS in CAR T therapy in the near future.

## Supporting information

S1 Text The optimized FMC63-28z sequence. (PDF)

S1 Fig Effects of PSL, TO-207, and tocilizumab on osteopontin production by CD14^+^ cells. (PDF)

S2 Fig Effect of tocilizumab on monocyte-derived pro-inflammatory cytokines. (PDF)

S3 Fig Effect of anakinra on monocyte-derived pro-inflammatory cytokines. (PDF)
