# Supplementary material for: The novel multi-cytokine inhibitor TO-207 specifically inhibits pro-inflammatory cytokine secretion in monocytes without affecting the killing ability of CAR T cells
Source: PLoS One. 2020 Apr 22;15(4):e0231896. doi: 10.1371/journal.pone.0231896 (PMC7176125; doi:10.1371/journal.pone.0231896)
Supplement: S1 Text — (PDF) [file pone.0231896.s001.pdf]

The optimized FMC63-28z sequence

cgagaattcgccgccaccATGCTTCTCCTGGTGACAAGCCTTCTGCTCTGTGAGTTACCACACCC
AGCATTCCTCCTGATCCCAGACATCCAAATGACACAGACCACATCCTCTCTCTCTGCCTCC
CTTGGCGACAGAGTAACAATAAGTTGCCGCGCCTCCCAGGATATTTCAAAGTACCTCAATT
GGTACCAACAAAAGCCAGATGGTACAGTCAAACTTTTGATCTACCACACTTCAAGGCTTCA
TTCTGGGGTACCCAGCCGATTCAGTGGGTCCGGAAGCGGAACCGATTACTCTCTTACAAT
ATCAAATCTTGAACAGGAAGACATTGCTACCTATTTTTGTCAACAAGGGAATACTCTCCCA
TACACCTTTGGCGGAGGTACCAAGTTGGAAATCACGGGCAGCACCAGTGGGAGCGGTAA
GCCGGGCTCTGGGGAAGGGTCTACTAAGGGAGAAGTAAAGTTGCAGGAAAGTGGTCCTG
GATTGGTAGCGCCAAGTCAATCACTCTCAGTAACGTGTACTGTATCAGGAGTTAGCCTGC
CGGACTACGGTGTGTCCTGGATTAGGCAACCTCCGCGCAAAGGACTTGAGTGGCTTGGG
GTGATATGGGGTTCTGAGACGACATACTACAACAGTGCGTTGAAATCCAGACTGACTATC
ATTAAGGACAATTCCAAATCCCAGGTGTTCTTGAAGATGAACAGCCTTCAGACTGACGATA
CTGCCATATACTATTGTGCCAAGCACTACTATTATGGGGGGAGCTATGCAATGGATTACTG
GGGGCAGGGAACAAGCGTGACTGTTTCAAGCgcggccgcAATTGAAGTTATGTATCCTCCTC
CTTACCTAGACAATGAGAAGAGCAATGGAACCATTATCCATGTGAAAGGGAAACACCTTTG
TCCAAGTCCCCTATTTCCCGGACCTTCTAAGCCCTTTTGGGTGCTGGTGGTGGTTGGGGG
AGTCCTGGCTTGCTATAGCTTGCTAGTAACAGTGGCCTTTATTATTTTCTGGGTGAGGAGT
AAGAGGAGCAGGCTCCTGCACAGTGACTACATGAACATGACTCCCCGCCGCCCCGGGCC
CACCCGCAAGCATTACCAGCCCTATGCCCCACCACGCGACTTCGCAGCCTATCGCTCCA
GAGTGAAGTTCAGCAGGAGCGCAGACGCCCCCGCGTACCAGCAGGGCCAGAACCAGCT
CTATAACGAGCTCAATCTAGGACGAAGAGAGGAGTACGATGTTTTGGACAAGAGACGTGG
CCGGGACCCTGAGATGGGGGGAAAGCCGAGAAGGAAGAACCCTCAGGAAGGCCTGTAC
AATGAACTGCAGAAAGATAAGATGGCGGAGGCCTACAGTGAGATTGGGATGAAAGGCGA
GCGCCGGAGGGGCAAGGGGCACGATGGCCTTTACCAGGGTCTCAGTACAGCCACCAAG
GACACCTACGACGCCCTTCACATGCAGGCCCTGCCCCCTCGCggatccgga

gaattc: *Eco*RI site
gccgccacc: Kozak sequence
gcggccgc: *Not*I site
ggatcc: *Bam*HI site
