# Supplementary material for: The novel multi-cytokine inhibitor TO-207 specifically inhibits pro-inflammatory cytokine secretion in monocytes without affecting the killing ability of CAR T cells
Source: PLoS One. 2020 Apr 22;15(4):e0231896. doi: 10.1371/journal.pone.0231896 (PMC7176125; doi:10.1371/journal.pone.0231896)
Supplement: S1 Fig — (PDF) [file pone.0231896.s002.pdf]

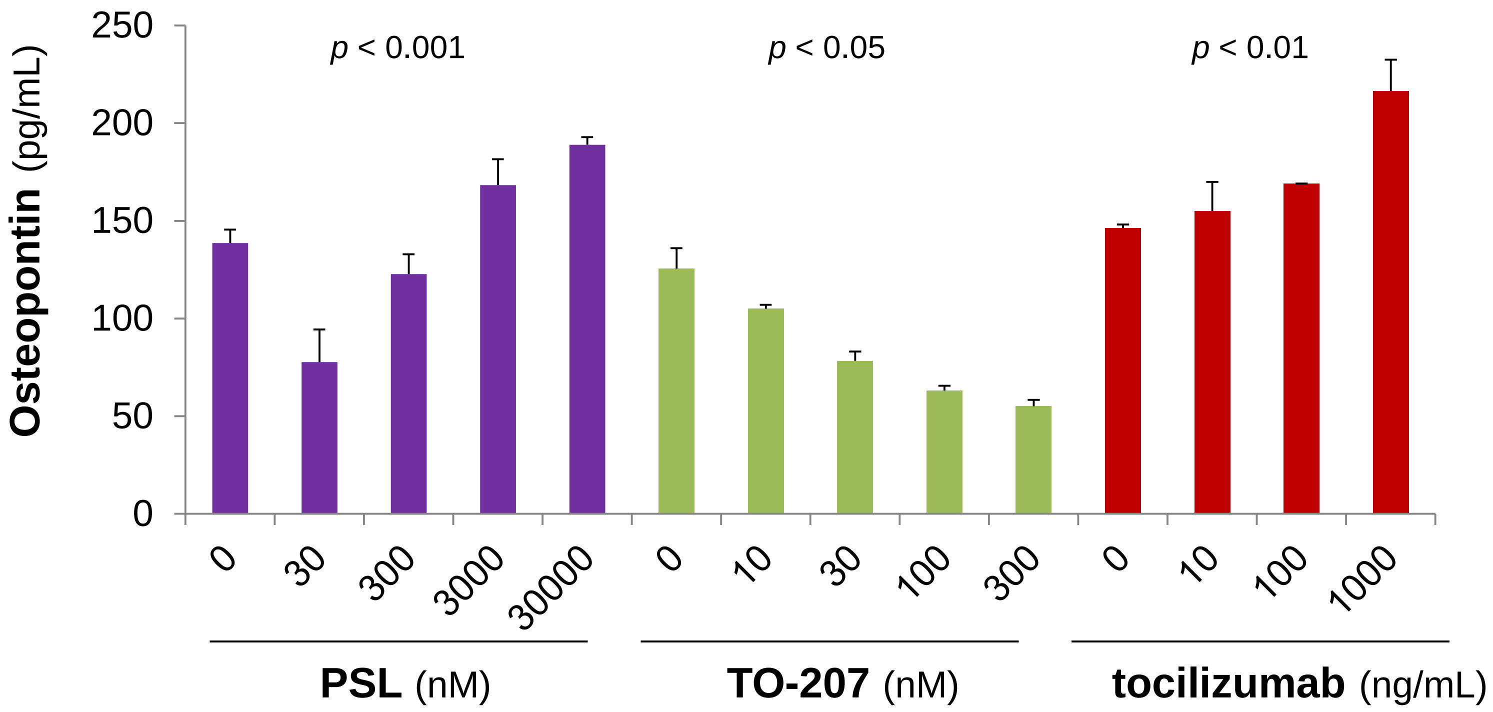

S2 Fig. Effects of PSL, TO-207, and tocilizumab on osteopontin production by $CD14^+$ cells. Peripheral blood $CD14^+$ cells (1×10^5) were seeded in a 96-well plate and incubated with PSL, TO-207, or tocilizumab for 30 min. The cells were then stimulated with 500 ng/mL LPS for 6 h. The supernatants were recovered, and osteopontin levels were determined by ELISA. The error bars represent standard errors (SEs) from three independent experiments. The linear dose–response relationship was assessed using log-transformed dose values (to the base 10) in a mixed model, in which the zero dose was replaced by the log (minimal dose) – 1. $P < 0.05$ was considered statistically significant.
