# Supplementary material for: The novel multi-cytokine inhibitor TO-207 specifically inhibits pro-inflammatory cytokine secretion in monocytes without affecting the killing ability of CAR T cells
Source: PLoS One. 2020 Apr 22;15(4):e0231896. doi: 10.1371/journal.pone.0231896 (PMC7176125; doi:10.1371/journal.pone.0231896)
Supplement: S2 Fig — (PDF) [file pone.0231896.s003.pdf]

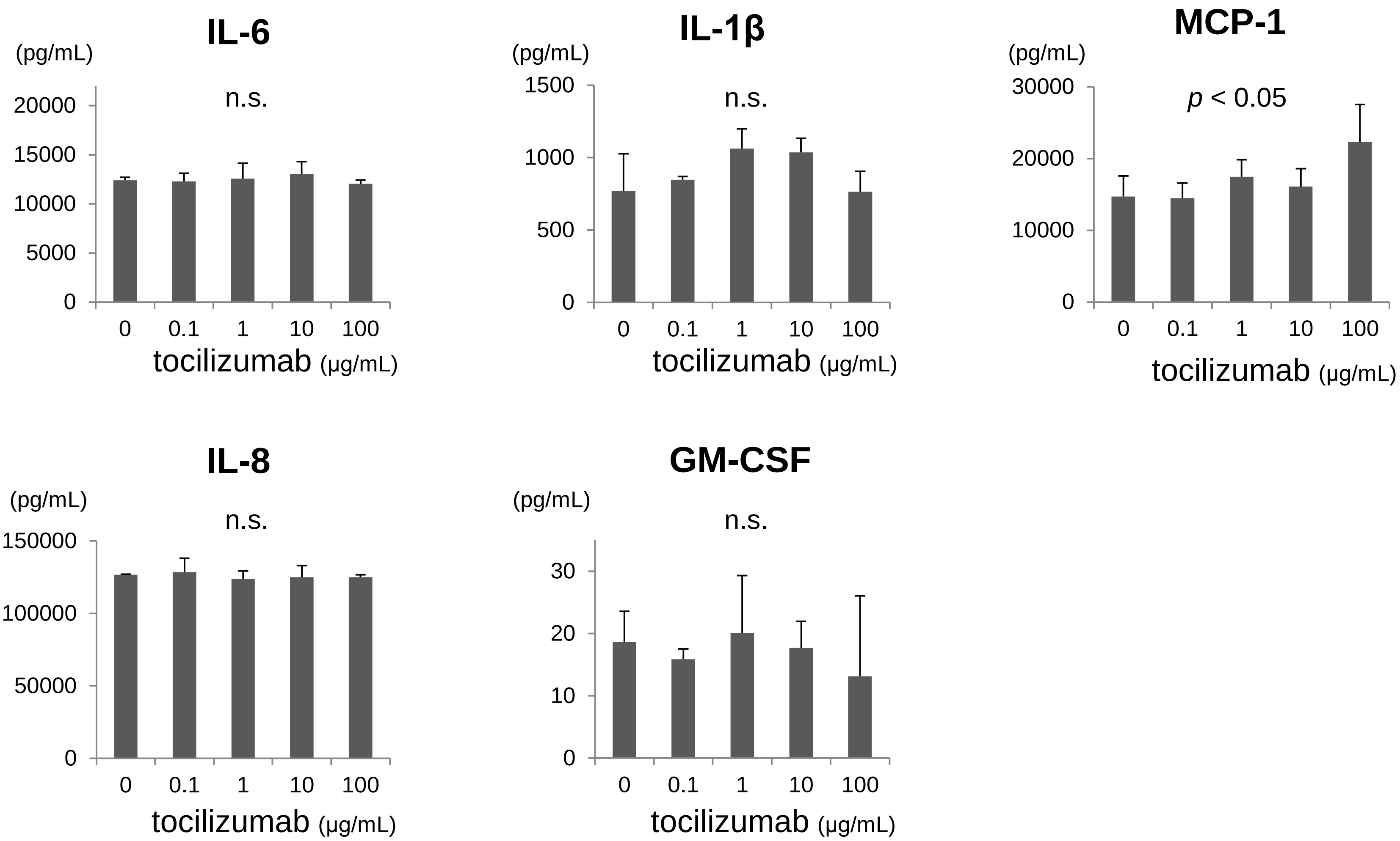

S3 Fig. Effect of tocilizumab on monocyte-derived pro-inflammatory cytokines. K562/CD19 cells (3×10^3), CAR-T cells (1.5×10^4), and $CD14^+$ cells (1.5×10^4) were co-cultured in a 96-well plate with different concentrations of tocilizumab (humanized monoclonal antibody against the IL-6 receptor). After 72 h, the culture supernatants were recovered, and cytokine levels were determined. The error bars represent SEs from three independent experiments. The linear dose–response relationship was assessed using log-transformed dose values (to the base 10) in a mixed model, in which the zero dose was replaced by the log (minimal dose) – 1. $P < 0.05$ was considered statistically significant. n.s.: not significant.
