# Supplementary material for: The novel multi-cytokine inhibitor TO-207 specifically inhibits pro-inflammatory cytokine secretion in monocytes without affecting the killing ability of CAR T cells
Source: PLoS One. 2020 Apr 22;15(4):e0231896. doi: 10.1371/journal.pone.0231896 (PMC7176125; doi:10.1371/journal.pone.0231896)
Supplement: S3 Fig — (PDF) [file pone.0231896.s004.pdf]

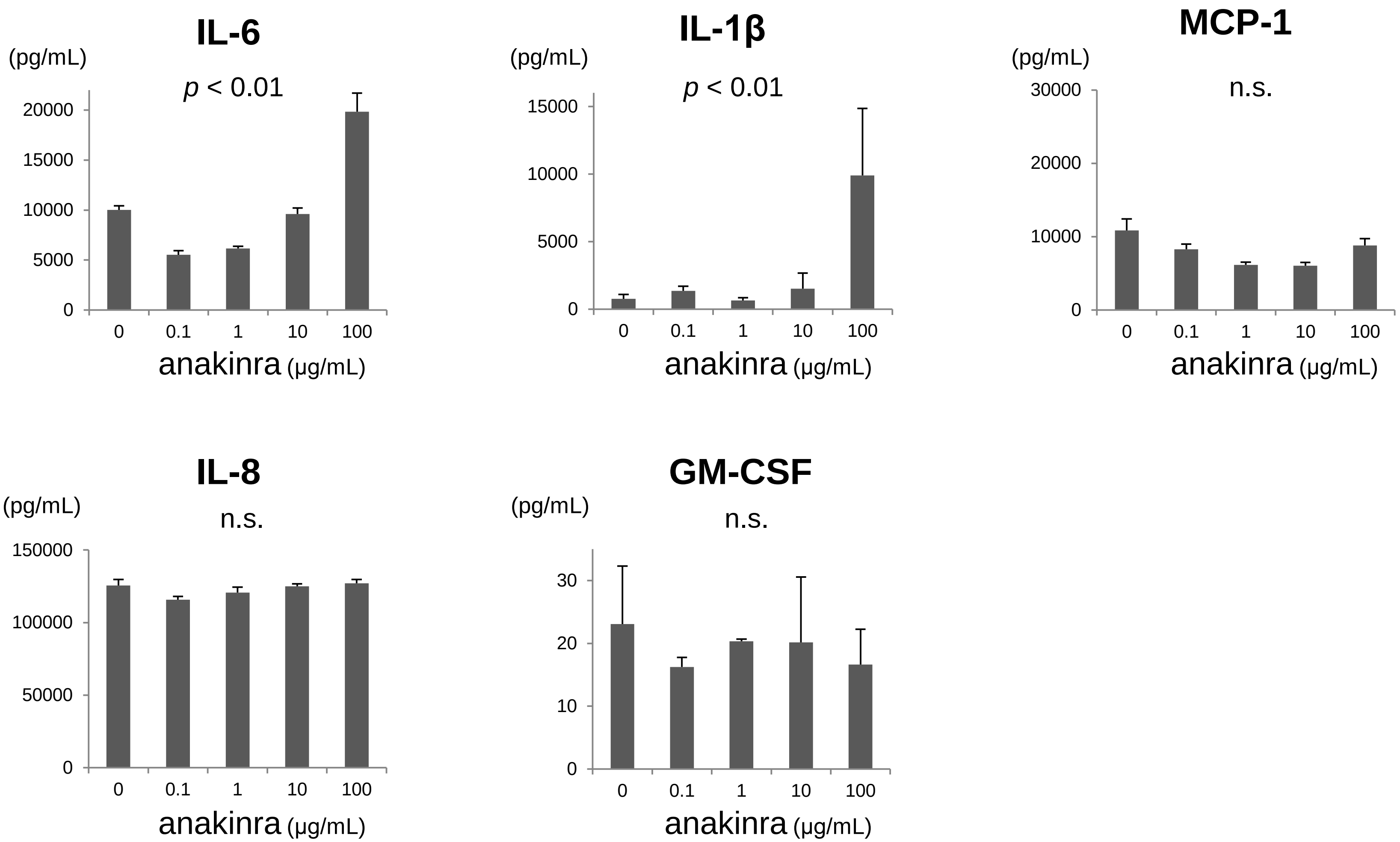

S4 Fig. Effect of anakinra on monocyte-derived pro-inflammatory cytokines. K562/CD19 cells (3×10^3), CAR-T cells (1.5×10^4), and $CD14^+$ cells (1.5×10^4) were co-cultured in a 96-well plate with different concentrations of anakinra, a recombinant human IL-1 receptor antagonist. After 72 h, the culture supernatants were recovered, and cytokine levels were determined. The error bars represent SEs from three independent experiments. The linear dose–response relationship was assessed using log-transformed dose values (to the base 10) in a mixed model, in which the zero dose was replaced by the log (minimal dose) – 1. $P < 0.05$ was considered statistically significant. n.s.: not significant.
